# Effect of N_2_ and H_2_ plasma treatments on band edge emission of ZnO microrods

**DOI:** 10.1038/srep10783

**Published:** 2015-06-01

**Authors:** Joana Rodrigues, Tiago Holz, Rabie Fath Allah, David Gonzalez, Teresa Ben, Maria R. Correia, Teresa Monteiro, Florinda M. Costa

**Affiliations:** 1 Departmento de Física & I3N, Universidade de Aveiro, Campus Universitário de Santiago, 3810-193 Aveiro, Portugal; 2 Departamento de Ciencia de los Materiales e I. M. y Q. I, Facultad de Ciencias, Universidad de Cádiz, E-11510 Puerto Real, Spain

## Abstract

ZnO microrods were grown by laser assisted flow deposition technique in order to study their luminescence behaviour in the near band edge spectral region. Transmission electron microscopy analysis put in evidence the high crystallinity degree and microrod’s compositional homogeneity. Photoluminescence revealed a dominant 3.31 eV emission. The correlation between this emission and the presence of surface states was investigated by performing plasma treatments with hydrogen and nitrogen. The significant modifications in photoluminescence spectra after the plasma treatments suggest a connexion between the 3.31 eV luminescence and the surface related electronic levels.

One-dimensional (1D) nanostructures have received most attention due to their excellent properties associated to their large surface area/volume ratio owing the possibility of nanoscale devices^1^. Among these 1D nano-objects, zinc oxide (ZnO) nanorods and nanowires are between the most studied structures in the last decades^2^. Part of the interest comes from the wide band gap of ZnO (~3.4 eV at room temperature (RT)^3^) and its high free exciton (FX) binding energy (60 meV^4^), which make this material a suitable host for many applications in optics and optoelectronics^5^,^6^.

The emission observed around 3.31 eV was many times associated to an unknown acceptor responsible for the non-reproducible p-type conductivity in this material^7^. However, the fact that this band also appears in nominally undoped n-type samples requires a more careful analysis. The 3.31 eV emission has been extensively studied in bulk^8^, thin films^9^ and nanostructures^10^, and has been associated to distinct recombination mechanisms such as donor-valence band transitions, electron-hole recombination from donor-acceptor pairs (DAP) transitions, electron-acceptor transitions, excitons bound to deep neutral acceptors, two electron satellite of donor bound excitons (D^0^X), longitudinal optical (LO) replicas of the D^0^X, among others, as previously reported^9^,^11^. Nowadays, the most accepted hypotheses for the nature of this band are the surface excitonic contribution^10^,^12^ or structural defects-related transitions^9^,^11^,^13^,^14^. Thonke and co-workers^9^,^13^,^15^ made an extensive investigation of the 3.31 eV emission in ZnO films and pillars and, using space resolved cathodoluminescence (CL), proved that in the case of their samples, this emission only appears in well-defined defect lines. Monochromatic CL images recorded at 3.31 eV allowed identifying basal plane stacking faults as the origin of this emission^9^,^13^,^15^.

A recent work published by Khranovskyy *et al*.^16^ reported the presence of basal plane stacking faults in wurzite ZnO nanowires that act like zinc blende quantum wells in a type II band alignment. These authors state that a emission observed at 3.329 eV (at 4 K) is a result of an indirect excitation transition from electrons in the condition band of the quantum well, which recombine with holes confined at the interface of the basal plane stacking faults. Similar results were observed for other semiconductors like in GaN^17^ and GaAs^18^ nanowires with wurzite/zinc blend heterostructures.

Another widely discussed hypothesis is the presence of surface states and excitons bounded to surface defects^10^,^12^. This argument is supported by the fact that this emission is frequently observed in samples with a high surface/volume ratio^19^. Fallert *et al*.^10^ reported a study where ZnO nanoparticles with different diameters were analysed by photoluminescence (PL). Those samples also exhibit a strong luminescence band around 3.31 eV and the exact position of this band slightly changes from sample to sample. The authors showed that decreasing the size of the nanoparticles, the intensity of the emission around 3.31 eV increases in comparison with the D^0^X emission^10^. Considering that the 3.31 eV/D^0^X intensities ratio depends on the surface/volume ratio and it is proportional to the inverse of the particle size, they conclude that the luminescence is associated mainly with states present at the particle’s surface suggesting a bound-exciton-like transition model for the recombination^10^. In the works performed by Tainoff *et al*.^11^,^14^ the authors studied different types of samples (single crystals, nanorods and nanoparticles) and concluded that the 3.31 eV emission is composed by an overlap of different processes, namely a defect-related transition, since the 3.31 eV appeared in the PL spectra of the nanorods even when the FX emission was not observed.

Aiming to contribute to clarify the origin of this emission line, we report a study on the near band edge luminescence of ZnO microrods grown by laser assisted flow deposition (LAFD) that were subject to post-growth hydrogen and nitrogen plasma treatments. Besides the usual free and donor bound exciton transitions observed in high optical quality ZnO samples^4^,^20^, the 3.31 eV and their phonon replicas were also identified. The latter was found to be strongly sensitive to the plasma treatments and excitation density, suggesting a correlation between the 3.31 eV luminescence and the surface related electronic levels.

## Experimental details

**Figure 1:**
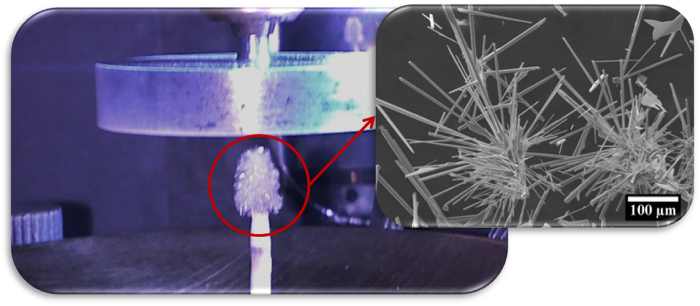
ZnO crystals formed by the LAFD at the precursor rod tip. The inset shows a high amplification SEM image of an agglomerate of as-grown ZnO microrods.

ZnO microcrystals were grown by LAFD technique performed on a modified laser floating zone growth chamber which comprises a 200 W CO_2_ laser (Spectron) coupled to a reflective optical set-up producing a circular crown-shaped laser beam. The beam is focused on the tip of the extruded cylindrical rods, previously prepared by mixing the ZnO powders (AnalaR, 99.7%) with polyvinyl alcohol (PVA, 0.1 g.ml-1, Merck), as reported elsewhere^21^,^22^,^23^. Different ZnO morphologies (microrods, nanoparticles and tetrapods) can be obtained during the growth as a result of different kinetics/thermodynamics local conditions verified in different regions of the growth chamber. In the region near the top of the precursor, owing to the high temperatures and permanent air circulation, the microrods grow directly on the precursor rod, as can be seen in Fig. 1. The growth was carried out in air during 3 minutes at 35 W.

The morphology of ZnO crystals was characterized by scanning electron microscopy, SEM (Hitachi SU-70) with a operating voltage of 25 kV. The sample microstructure was characterized by conventional transmission electron microscopy (TEM), high resolution TEM (HRTEM) and high angle annular dark field scanning TEM (HAADF-STEM). All the measurements were performed in JEOL 1200 EX and JEOL 2010F microscopes operating at 120 and 200 kV, respectively. The samples were prepared for TEM studies by a scraping method where the microrods were removed from the precursor rod, dispersed, and dropped on a TEM grid.

Steady state macro-PL measurements were performed using the 325 nm (3.8 eV) line from a cw He-Cd laser with a beam spot of ∼1 mm and an excitation power density less than 0.6 W.cm^−2^, as excitation source. The analysis was carried out under a 90^o^ geometry. The measurements were realized on samples comprised by several random oriented microrods glued in a carbon tape and attached to a cold finger of a closed-cycle helium cryostat. The sample temperature was controlled in a range from 14 K to RT. The luminescence was measured using a dispersive system SPEX 1704 monochromator (1 m, 1200 grooves.mm^−1^) fitted with a cooled Hamamatsu R928 photomultiplier tube. Macro-PL provides global information over the whole sample, illuminating a great number of microrods during the experimental acquisition. Since these rods are randomly oriented regarding the illumination, the luminescence was collected from both top and side planes of the ZnO rods. In all the cases (treated and untreated samples), approximately the same amount of microrods was used for the PL measurements. For each sample, different laser spots were recorded in order to check the luminescence homogeneity of the samples.

Hydrogenation was performed during 30 minutes in a commercial microwave (2.45 GHz) plasma chemical vapour deposition (MPCVD) system, ASTeX PDS-18, using H_2_ as the only feed gas. Hydrogen plasma treatments were conducted using two sets of parameters. In the first set 1500 W of microwave power, total pressure of 70 Torr and H_2_ flow of 250 ml.min^−1^ were used. In the second set of experiments the microwave power and total pressure were raised to 2000 W and 80 Torr respectively, keeping the other parameters as in the first step. The sample hydrogenated in the conditions of the first step (sample#2, as characterized in Table 1) was then treated at 700 °C during 5 h. Nitrogen plasma treatments were conducted using the same MPCVD reactor. The treatment was performed during 15 minutes with a N_2_ flow of 100 ml.min^−1^. As in the case of the hydrogenation, two sets of parameters were used. In the first set of experiments a microwave power of 900 W and a total pressure of 20 Torr were used. In the second set the power was fixed at 1000 W and the total pressure was raised to 40 Torr. The sample characteristics are listed in Table 1.

## Results and discussion

**Figure 2:**
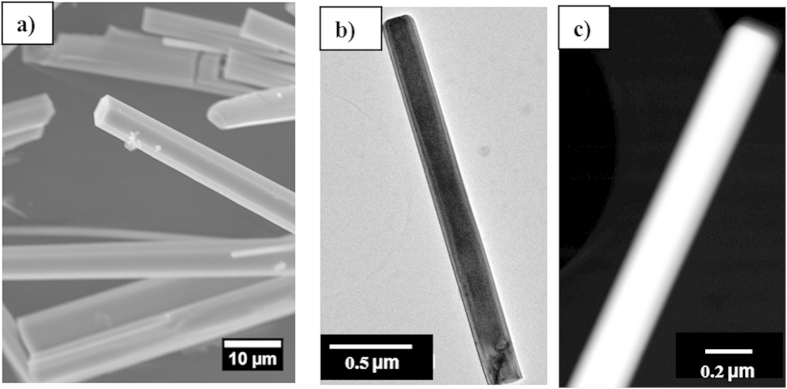
**(a)** SEM image of the ZnO microrods. (**b**)TEM and (**c**) HAADF-STEM of a ZnO microrod scraped from the precursor rod and dispersed on a TEM grid.

**Figure 3:**
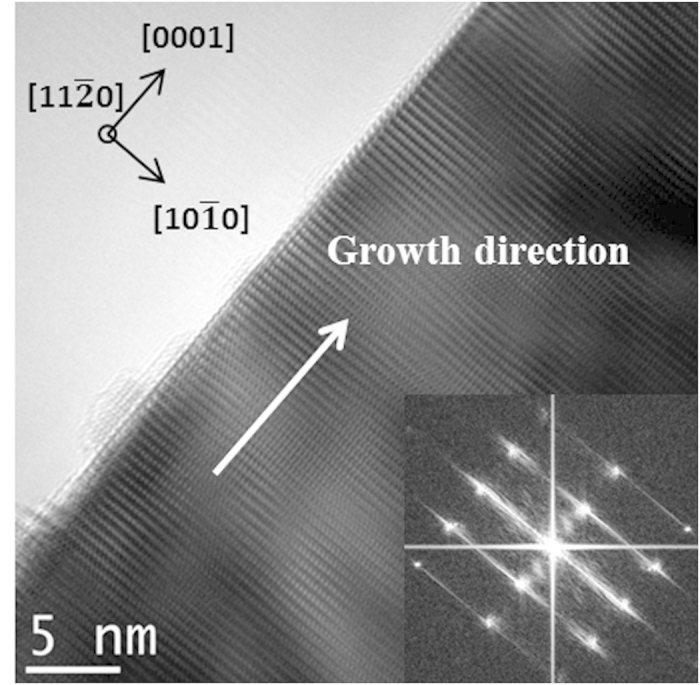
HRTEM image and the corresponding FFT of a single ZnO microrod confirming c-axis orientation.

Figure 2a shows a SEM image of the produced ZnO crystals. These samples exhibit a high aspect ratio with a hexagonal cross section. The rods are randomly oriented and their density depends on the region of the sample. A study of a significant number of scrapped microrods was carried out by TEM techniques (see an example in Fig. 2b). The analyses showed that the lengths of the microrods are in the range of several of μm with diameters around 200-500 nm. In addition, HAADF-STEM imaging (Fig. 2c) revealed a lack of intensity contrasts inside the ZnO microrods and pointed to a homogenous composition along the growth and radial directions. In order to analyse the crystal structure and the presence of extended defects by TEM, the microrods were tilted to align the electron beam with their 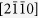 crystallographic direction. HRTEM analysis and the corresponding Fast Fourier Transform (FFT) (see an example in Fig. 3) confirmed that all the studied microrods grew along the <0001> direction. No dislocations or basal stacking faults perpendicular to the general [0001] growth direction were found along the entire microrod length, even in the apex. Moreover, the lateral walls of the microrods appear almost atomically flat without roughness or a shell structure.

**Figure 4:**
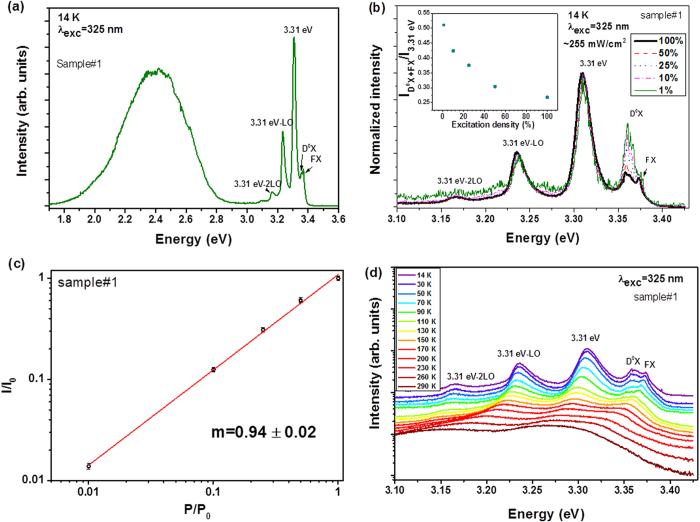
**(a)** Low temperature PL of the near band edge emission of the ZnO microrods grown by LAFD (sample#1). (**b**) Excitation density dependence of the near band edge emission recorded at 14 K. The inset depicts the ratio between the D^0^X + FX and 3.31 eV emission intensity as a function of excitation power. (**c**) log (I/I_0_)-log (P/P_0_) plot for the 3.31 eV emission. (**d**) Temperature dependence of the near band edge emission.

PL measurements performed at low temperature (14 K) (Fig. 4a) revealed the presence of an intense broad band in the green spectral region related to deep level defects^24^,^25^,^26^. In the near band edge region the emission is dominated by a line at ~3.31 eV as observed by its highest intensity when compared with the other emission lines. The emission due to the free exciton (FX) can be found at ~3.37 eV and the D^0^X lines are also present at ~3.36 eV. The energy separation between the FX and the 3.31 eV emission is ~64 meV, which is in close agreement with the values reported in the literature^19^,^27^,^28^. The emission observed at ~3.24 eV is most likely due to the first longitudinal optical (LO) phonon replica of the 3.31 eV line, and a second phonon replica can also be observed at ~3.16 eV, each with an energy separation of ~70 meV.

As aforementioned, a well-accepted origin for the 3.31 eV optical centre was related with the presence of basal plane stacking faults^9^,^13^,^15^. However in the case of these samples produced by LAFD, the TEM analysis showed that the as-grown microrods did not reveal any evidence of dislocations or stacking faults regardless of the emission visible at 3.31 eV. Thus, a different origin should be responsible for this emission band.

As in the case of previous works reported by other authors^19^, in our samples excitation density studies revealed a strong dependence of the emission intensity with the excitation power (Fig. 4b). The relative intensity of the emission related with the D^0^X increases in comparison with the 3.31 eV when the excitation density decreases, as shown in the inset of Fig. 4b. A study of the PL emission as a function of the illumination time was carried out during 3 hours to understand the effect of the local heating caused by the laser beam in the sample (not shown). No changes in the relative intensity between the 3.31 eV and D^0^X lines were observed, evidencing that the behaviour observed with the excitation density is not related with a local heating. The presence of defects caused by photon irradiation can be ruled out since the emission intensity is reversible. Similar behaviour were attributed to the saturation of the D^0^X recombination with increasing excitation density^19^. It is important to note that no shift was observed in the peak position of the 3.31 eV emission with decreasing excitation density, eliminating the possibility of a DAP recombination. The assumption of having a localized state is compatible with the dependence on the excitation intensity also observed by Schneider *et al*.^19^. As a function of the excitation density, the PL intensity of the 3.31 eV line (Fig. 4c) can be well fitted to a power law, 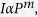 where *I* is the luminescence intensity, *P* is the excitation power and *m* is a parameter that represents the slope in a log-log representation of *I* and *P*. The 3.31 eV line was well fitted by a Lorentzian expression. The analysis reveals a slope just below the unity (0.94 ± 0.02). According to T. Schmidt *et al*.^29^, *m* < 1 proposes that the nature of the radiative transitions involves DAPs or free-to-bound carrier recombination. However, the presence of DAP was already ruled out, suggesting that in the present case we are in the presence of a free-to-bound transition. Temperature dependence of the near band edge emission is represented in Fig. 4d. Increasing the temperature promotes the dissociation of the bound excitons and a redshift is observed for the FX, 3.31 eV line emission and its replicas. This behaviour is similar to the one observed by Schirra *et al*.^9^, where the authors associated the 3.31 eV emission to a free-to-bound transition involving the recombination of an electron from the conduction band with a hole bound to an acceptor state (e,A_0_).

The influence of the surface becomes important when dealing with samples having high surface to volume ratio as is the case of the microrods produced in this work. The presence of surface states due to dangling bonds, point defects or surface adsorbed species must be considered as potential influencers on the material’s optical properties.

**Figure 5:**
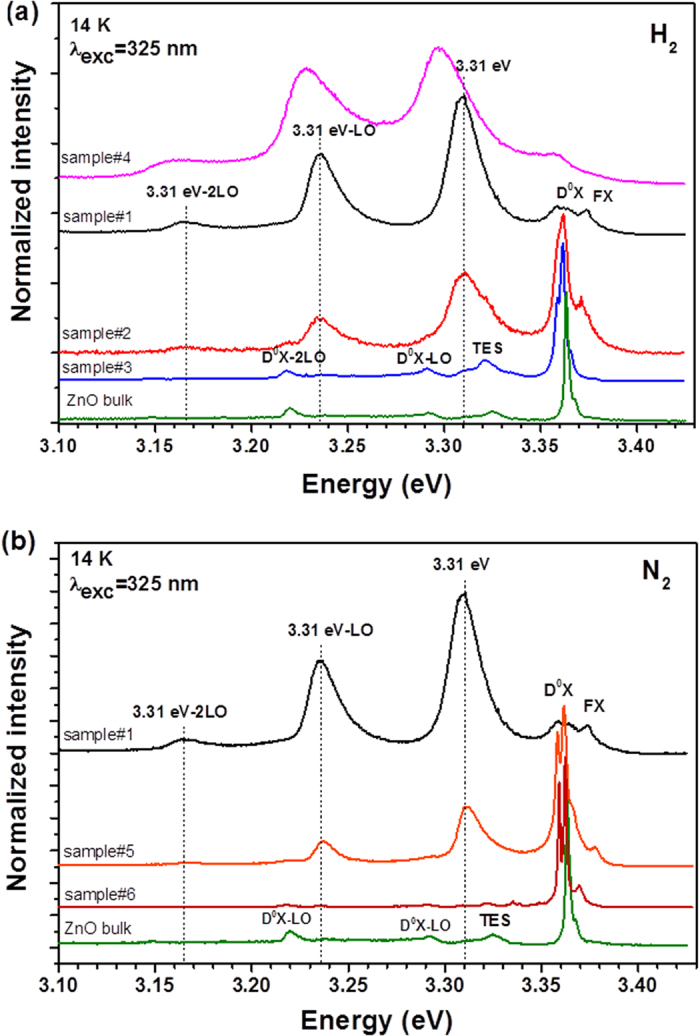
Comparison between the PL emission before and after the treatment with (**a**) H and (**b**) N plasma. The luminescence spectrum of a bulk sample was include for comparison.

In order to understand the nature of the 3.31 eV emission, hydrogen and nitrogen plasma passivation treatments were performed in the ZnO microrods grown by LAFD. The spectra recorded before and after the treatments are shown in Fig. 5. After the first hydrogenation treatment (sample#2) the intensity of the 3.31 eV line decreased comparing to the D^0^X line. The latter became the dominant emission in the near band edge region. Since a decrease in the intensity of the line in question was observed, a higher plasma power was used (sample#3) with the purpose of further reduction. In the case of sample#3, the line observed at 3.31 eV was suppressed, as well as its phonon replicas at ~3.24 eV and ~3.16 eV. Instead, sample#3 clearly evidences the two electron satellites (TES) lines at ~3.32 eV and the phonon replicas of the D^0^X transitions ∼70 meV apart from the main transition at ~3.36 eV. The PL spectrum of sample#3 resembles the one obtained for a bulk sample, as can be observed in Fig. 5a. Therefore, passivation with H_2_ plasma leads to a luminescence emission similar to the one obtained for ZnO bulk sample. As H_2_ is known to be a fast interstitial diffuser in ZnO (0.2 cm^2^/s^30^) even at low temperatures (100 °C)^31^ the performed H_2_ plasma treatments should promote the incorporation of hydrogen throughout the entire volume of the ZnO microrods leading to an increase in the intensity of the H-related D^0^X, the I_4_ line^4^ peaked at ~3.362 eV, as identified in Fig. 5a. It is worth to mention that at the present plasma conditions a temperature around 900 °C is expected inside the reactor. Several works reported the enhancement of the NBE emission of ZnO after the treatment with H_2_ plasma^32^,^33^,^34^. Therefore, the incorporation of hydrogen as a dopant into the ZnO matrix must be taken into account to explain the increasing of the D^0^X emission intensity after the passivation. All the incorporated hydrogen by the plasma treatment is expected to be removed after a thermal treatment with temperatures higher than 500–600 °C^31^. Sample#3 was further submitted to a thermal annealing at 700 ^o^C in air during 5 hours. As evidenced in Fig. 5a, after this treatment (sample#4) the D^0^X emission is reduced and the emission around 3.31 eV became the dominant once again, despite of a slightly shift to lower energies, which could be related with the thermal expansion of the lattice during the annealing. The recovering of the emission is an indication that the H_2_ concentration after the annealing was reduced as identified by the strong decrease of the D^0^X line intensity. As previously reported^32^, the H_2_ plasma treatment leads to a reversible process.

Figure 5b depicts the spectra of the N_2_ treated samples. A decrease of the relative intensity of the 3.31 eV line regarding the D^0^X emission is clearly observed after the first treatment (sample#5). The treatment carried out with higher N_2_ plasma power (sample#6) revealed a suppression of the 3.31 eV emission. In both samples a high resolution of the splitted D^0^X lines is observed. As nitrogen is a slow diffuser in ZnO^35^, surface treatments with N_2_ plasma were attempted in the ZnO microrods in order to infer about the presence of surface states and their relationship with the 3.31 eV emission. Børseth *et al*.^35^ reported a diffusivity up to 10^−15^ cm^2^/s based on an estimated maximum diffusion length of 10 nm after 1 h annealing at 1000 °C. As in the case of the hydrogenation, the estimated temperature inside the reactor chamber is higher than 700 °C, so it is expected that most of the nitrogen remains at the surface of the ZnO microrods. Since the passivation with nitrogen acts on the surface states, the suppression of the band observed at 3.31 eV after the second plasma treatment (sample#6) suggests that the surface states are in the origin of the mentioned emission.

**Figure 6:**
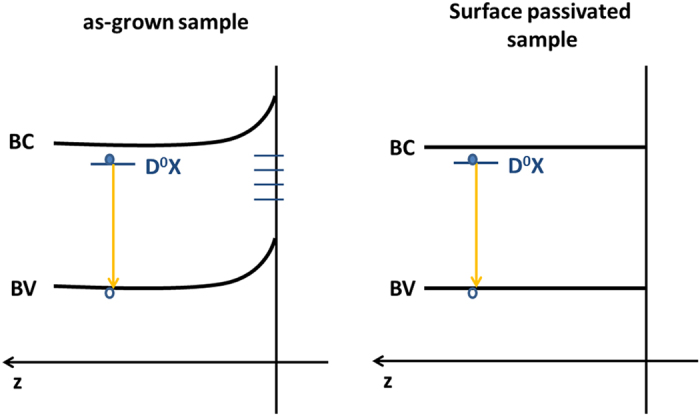
Schematic representation of the proposed energy band diagram of the surface of the ZnO microrods before and after passivation.

Figure 6 depicts a schematic representation of a proposed model for the energy band diagram of the surface of the ZnO microrods before and after passivation. The presence of surface states due to dangling bonds, point defects or surface adsorbed species at the ZnO surface causes a band bending of the energy bands near the ZnO surface, which leads to the capture of charged carriers near the surface and the generation of a surface charge^36^. The formation of this depletion region will lead to modifications in the recombination processes, which becomes particularly important for samples with a high surface to volume ratio. If these defects are optically active, new emission may arise, dominating over the bulk luminescence. When the surfaces are passivated, eliminating dangling bonds or surface adsorbed species, a strongly reducing of these surface states occurs, the bending of the bands can be almost neglected and the bulk’s recombination processes dominate.

**Figure 7:**
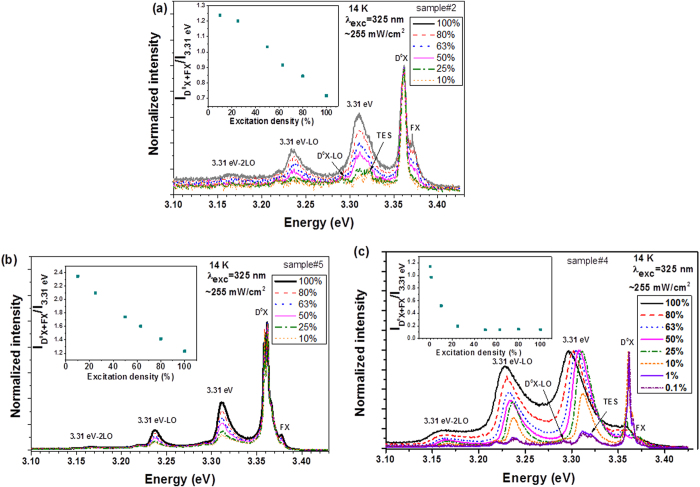
Excitation density dependence of the PL for (**a**) sample#2, (**b**) sample#5 and (**c**) sample#4. The insets correspond to the ratio between the integrated intensity of the D^0^X + FX and the 3.31 eV emission lines for each sample.

Excitation density studies were performed in all the treated samples (Fig. 7) revealing an accentuated reduction of the 3.31 eV PL intensity with decreasing excitation density, as observed for the case of the samples #2 (Fig. 7a), #5 (Fig. 7b) and #4 (Fig. 7c). For the lowest excitation power, the mentioned band is decomposed into two emission lines, one at ~3.32 eV due to the TES recombination and another at ~3.31 eV. In the samples #3 and #6 the later emission was almost suppressed and consequently no significant changes with the decreasing of the excitation density were observed (not shown). This observed dependence on the excitation density of the intensity of the dominant PL lines (3.31 eV versus D^0^X) indicates that, as for the case of sample#1, a saturation effect of the D^0^X recombination leading to the change in the relative intensity of both lines. Comparing the ratio between the intensities of the D^0^X + FX and 3.31 eV line emissions as a function of the excitation density for the plasma treated samples, a ratio decrease with increasing excitation power is observed. In the case of the sample treated with H_2_ plasma the decrease was about 56% while for the sample treated with N_2_ a decrease of 52% was found. These values point to an analogous behaviour for both treatments, which can indicate that the phenomena involved in the passivation are similar and most of the H is located at the surface of the microrods instead of the bulk. However, this small difference can imply that the N_2_ plasma is more efficient in the passivation of the surface defects. For sample#4 a red shift of the 3.31 eV line with increasing excitation density is observed. This behaviour can be attributed to possible changes at the surface charge density after the annealing at high temperature (700 ^o^C) due to the presence of different species adsorbed at surface. It is well known that after annealing at this kind of temperatures re-adsorption of species (like the case of OH-groups) is expected when the samples are exposed to the ambient^36^. This fact associated with the number of charge carriers that are being generated under the different excitation powers can conduct to modifications in the band bending. It is likely that a decrease in the band bending energy promotes the observed red shift. It is also convenient to mention that for low excitation powers an inversion of the D^0^X and 3.31 eV line emissions relative intensities is observed, which did not occurred for the as-grown sample. This fact can be explained by the incorporation of H ions inside the ZnO matrix promoted by both passivation and annealing treatments, leading to an increase of the D^0^X emission when compared with the as-grown case.

Despite the unknown nature of the defects, that generate the 3.31 eV band in the studied samples, the performed passivation treatments suggest that surface defects cannot be ruled out on the band recombination model. The excitation density dependence of the luminescence data clearly evidences that for the highest excitation densities the generated free carriers are predominantly captured by the defects from where 3.31 eV emission arises, as inferred from the passivation studies. An effective control of the 3.31 eV PL relative intensity can be obtained by controlling the excitation density.

## Conclusions

ZnO microrods were grown along the <0001> growth direction by the LAFD technique. Despite the fast growth rate, TEM analysis confirmed the high crystallinity of the samples and the absence of dislocations or stacking faults in the analysed structures. The emission at higher energies is dominated by the 3.31 eV band. Plasma treatments (H_2_ and N_2_) were performed in order to study the nature of the defects associated with this 3.31 eV emission and it was observed that after the treatments significant changes occurred in the PL intensity in this spectral region. The 3.31 eV band was suppressed for the samples treated with a higher power for both plasma natures. The suppression observed with the N_2_ plasma suggests that the emission could be related with the presence of surface states in nature, since nitrogen has a low diffusivity in ZnO at the temperature present inside the reactor and it is expect that most of it remains at the surface. Furthermore, it was found that an effective control of the PL intensity of the 3.31 eV versus D^0^X lines can be reached by considering the effects of the excitation intensity on the free exciton capture.

## Additional Information

**How to cite this article**: Rodrigues, J. *et al*. Effect of N_2_ and H_2_ plasma treatments on band edge emission of ZnO microrods. *Sci. Rep*. **5**, 10783; doi: 10.1038/srep10783 (2015).

## Figures and Tables

**Table 1:** Plasma and annealing treatment conditions of each sample.

	MPCVD plasma treatment			Annealing treatment
Sample	Power (W)	Pressure (Torr)	Gas flow (ml.min^−1^)	
Sample#1	–	–	–	LAFD as-grown microrods
Sample#2	1500	70	H_2_ 250	–
Sample#3	2000	80	H_2_ 250	–
Sample#4	1500	70	H_2_ 250	700 °C/5 h
Sample#5	900	20	N_2_ 100	–
Sample#6	1000	40	N_2_ 100	–
